# Furosemide versus ethacrynic acid in pediatric patients undergoing cardiac surgery: a randomized controlled trial

**DOI:** 10.1186/s13054-014-0724-5

**Published:** 2015-01-07

**Authors:** Zaccaria Ricci, Roberta Haiberger, Chiara Pezzella, Cristiana Garisto, Isabella Favia, Paola Cogo

**Affiliations:** Department of Cardiology and Cardiac Surgery, Pediatric Cardiac Intensive Care Unit, Bambino Gesù Children’s Hospital, IRCCS, Piazza S. Onofrio 4, 00165 Rome, Italy

## Abstract

**Introduction:**

Clinical effects of furosemide (F) and ethacrynic acid (EA) continuous infusion on urine output (UO), fluid balance, and renal, cardiac, respiratory, and metabolic function were compared in infants undergoing surgery for congenital heart diseases.

**Methods:**

A prospective randomized double-blinded study was conducted. Patients received 0.2 mg/kg/h (up to 0.8 mg/kg/h) of either F or EA.

**Results:**

In total, 38 patients were enrolled in the F group, and 36, in the EA group. No adverse reactions were recorded. UO at postoperative day (POD) 0 was significantly higher in the EA group, 6.9 (3.3) ml/kg/h, compared with the F group, 4.6 (2.3) ml/kg/h (*P* = 0.002) but tended to be similar in the two groups thereafter. Mean administered F dose was 0.33 (0.19) mg/kg/h compared with 0.22 (0.13) mg/kg/h of EA (*P* < 0.0001). Fluid balance was significantly more negative in the EA group at postoperative day 0: −43 (54) ml/kg/h versus −17 (32) ml/kg/h in the F group (*P* = 0.01). Serum creatinine, cystatin C and neutrophil gelatinase-associated lipocalin levels and incidence of acute kidney injury did not show significant differences between groups. Metabolic alkalosis occurred frequently (about 70% of cases) in both groups, but mean bicarbonate level was higher in the EA group: 27.8 (1.5) *M* in the F group versus 29.1 (2) m*M* in the EA group (*P* = 0.006). Mean cardiac index (CI) values were 2.6 (0.1) L/min/m^2^ in the F group compared with 2.98 (0.09) L/min/m^2^ in the EA group (*P* = 0.0081). Length of mechanical ventilation was shorter in the EA group, 5.5 (8.8) days compared with the F group, 6.7 (5.9) (*P* = 0.06). Length of Pediatric Cardiac Intensive Care Unit (PCICU) admission was shorter in the EA group: 14 (19) days compared with 16 (15) in the F group (*P* = 0.046).

**Conclusions:**

In cardiac surgery infants, EA produced more UO compared with F on POD0. Generally, a smaller EA dose is required to achieve similar UO than F. EA and F were safe in terms of renal function, but EA caused a more-intense metabolic alkalosis. EA patients achieved better CI, and shorter mechanical ventilation and PCICU admission time.

**Trial registration:**

Clinicaltrials.gov NCT01628731. Registered 24 June 2012.

## Introduction

Loop diuretics (LDs) are frequently administered to critically ill children to manage the high fluid load that is prescribed daily and to control fluid balance. LD pharmacologic action is exerted at the thick ascending limb of Henle loop by reversibly blocking one of the chloride-binding sites of the Na^+^/K^+^/2 Cl^−^ carrier. Consequently, reabsorption of filtered sodium is inhibited. This intense natriuresis promotes an increased diuresis [1]. In Pediatric Intensive Care Units (PICUs), LDs are routinely administered by either intermittent or continuous intravenous infusions [2]. The vast majority of pediatric cardiac surgery patients receive LDs in the preoperative, intraoperative, and postoperative phases. LDs are the mainstay of therapy in children with congenital heart disease (CHD), particularly in the near-postoperative phase: they are essential for the management of fluid loading and the leak syndrome occurring after cardiopulmonary bypass (CPB) and of the high fluid administration needed for postoperative therapies and nutrition [3]. Postoperative fluid overload in patients with congenital heart disease has been associated with increased occurrence of acute kidney injury (AKI), prolonged length of mechanical ventilation, worsened respiratory function, and delayed discharge from the PICU [4-7].

Timely treatment of fluid excess has never been prospectively examined: diuretics, however, are recommended for management of fluid overload in adults and children [8]. Furthermore, in patients with cardiac dysfunction, LDs are needed both for left ventricular failure (to decrease hypervolemia, ventricular filling pressures, and pulmonary congestion) and right ventricular failure (to control fluid accumulation and systemic venous congestion, both of which are causes of renal and splanchnic organ dysfunction) [9]. The presence of well-known side effects, however, including metabolic alkalosis, the risk of neurohormonal activation, systemic vasoconstriction, electrolyte disturbances, impairment of renal function, and perhaps, worse clinical outcomes, may lead to arguments against an aggressive diuretic approach in these patients [10]. It was previously shown that continuous infusion of furosemide causes a gradual increase in urine output (UO) that is significantly superior, in terms of total daily diuresis, to the unpredictable urine volume secondary to intermittent boluses [11]. Commonly administered LDs in PICUs are furosemide, bumetanide, and ethacrynic acid. In our institution, furosemide and ethacrynic acid are administered without any specific protocol. Although few studies exist on the relative pharmacokinetic properties of these two drugs [12], ethacrynic acid is considered to be 30% less potent than furosemide [12]. The exact effect on diuresis, the appropriate dose of these drugs’ continuous infusion, the difference between the pharmacologic effects of furosemide and ethacrynic acid, and their side effects have never been addressed previously in pediatric patients with CHD.

The aim of this study was to compare the clinical effects of a continuous infusion of furosemide with a continuous infusion of ethacrynic acid on UO, control of fluid balance, and on renal, cardiac, respiratory, and metabolic function in a population of infants undergoing surgery for CHD.

## Methods

A prospective randomized double-blind study (the Furocrynic Study) was conducted at the Pediatric Cardiac Intensive Care Unit (PCICU), Ospedale Bambino Gesù, Rome, Italy, from June 2012 to November 2013 (NCT01628731). Consecutive infants undergoing elective surgery for CHD and who had signs of fluid overload or leak syndrome, according to the attending physician at PCICU admission, were enrolled. To assess roughly fluid loading received in the operating room (apart from blood loss and transfusions), intraoperative fluid balance was assessed and indexed on preoperative patients’ weight ((intraoperative fluid in-intraoperative urine output)/patients’ body weight × 100). It is almost impossible to evaluate intraoperative blood loss precisely, because of suction into big reservoirs, blood collected into cell savers, and blood absorbed by dressings; furthermore, blood-derivate transfusion bag number is reported only in our clinical charts, avoiding any exact calculation of infused fluids. Exclusion criteria were the presence of known preoperative renal dysfunction (need for preoperative dialysis or presence of renal malformations), preoperative chronic (<14 days) LD administration, need for extracorporeal membrane oxygenation at PCICU admission, and time of admission to the PCICU >60 minutes. The study could be interrupted at any time if the attending physician recorded any sign of adverse reaction related to the diuretic infusion (allergy, hemodynamic instability, arrhythmias).

### Objectives

The primary objective of this study was to verify the superiority of ethacrynic acid compared with furosemide in improving patients’ postoperative UO during the first postoperative day (POD0). The primary end point was to assess the mean daily UO (expressed in ml/kg/h) in POD0 (from PCICU admission to 7 AM of the next morning).

Secondary objectives were (a) to verify the superiority of ethacrynic acid compared with furosemide in improving patients’ postoperative UO during the first 72 hours, including the mean daily UO (expressed in ml/kg/h) in the first and second PODs (not only POD0); (b) to compare the daily dose required by each LD to achieve respective UOs in the first 3 PODs by indexing mean UO over mean diuretic dose at the end of each POD; (c) to measure fluid balance in the first 72 hours in the two groups; (d) to compare renal function after infusion of ethacrynic acid and furosemide by evaluating the occurrence of acute kidney injury (AKI) and the mean levels of serum creatinine (sCr), cystatin C (CysC), and whole blood neutrophil gelatinase-associated lipocalin (NGAL) in the two groups; (e) to compare the occurrence of hypokalemia and metabolic alkalosis in the two groups; (f) to compare the effects of the two LDs on hemodynamic and respiratory functions by measuring mean cardiac index (CI), mean ratio of PaO_2_ over fractional inspired oxygen (PaO_2_/FIO_2_), mean length of mechanical ventilation, and mean length of PCICU admission (LOA).

To examine whether any association was present between diuretic dose and other variables (weight, Aristotle score, vasoactive inotrope score, CPB duration, length of mechanical ventilation, LOA, creatinine values, UO, UO/diuretic dose), we conducted a secondary analysis comparing two significantly different subgroups of our patients: those who interrupted (any) diuretic continuous infusion on POD1 (early stoppers,ESs) and those who continued a full continuous diuretic dose (0.2 mg/kg/h or more) up to POD3 (late stoppers, LSs).

### Interventions

Enrolled patients were randomized to receive either 0.2 mg/kg/h of furosemide or an equivalent dose of ethacrynic acid in a double-blind fashion. The attending physicians were allowed to increase the drugs up to a corresponding dose of 0.8 mg/kg/h (as done in clinical practice, according to institutional protocols). After 72 hours, data collection was interrupted, and the diuretic was administered openly. Physicians were permitted to decrease or stop the continuous infusion if clinically indicated, at any time after enrolment, during the studied period, or to convert the continuous infusion to intermittent boluses: however, they were required to specify the reason for interruption. The only clinical goal that clinicians were requested to target, if possible, was an even or negative fluid balance at POD0. Furthermore, bolus doses were always 1 mg/kg, and the attending physician prescribed the dosing interval (6, 8, or 12 hours). In all other cases, attending physicians were free to act according to everyday practice.

### Randomization procedure

After patient enrollment, a nurse who was not involved in the care of the study patient prepared the diuretic infusion in an effort to maintain blinding of the study drug to the bedside nurse and clinician. The drug was diluted to provide a consistent rate of 0.5 ml/hour and dose of 0.2 mg/kg/hour to all patients and held in black syringes labeled “diuretic study”. The allocation sequence was generated by a computerized random-number generator. If the attending physician wanted to administer the study drug intermittently, boluses were prepared blinded to the bedside nurse and clinician.

### Data collection

Demographic data, diagnosis, type and risk of surgery (Aristotle score), and intraoperative information (CPB and cross-clamp length, need for deep hypothermic circulatory arrest, delayed sternal closure) were recorded at patient enrollment. Each enrolled patient had the same set of data available at every time point (PCICU arrival, POD0, POD1, POD2): hemodynamic data (heart rate; systolic, diastolic, and mean arterial pressure; left atrial pressure; central venous pressure; cardiac index), laboratory data (sCr, CysC, NGAL), blood gas analysis data (pH, SaO_2_, SvO_2_, bicarbonate concentration, base excess, lactates), and the amount of administered vasoactive drugs, including vasoactive inotropic score (VIS). PaO_2_/FIO_2_ was recorded at all time points. At POD0, POD1, and POD2 UO, fluid balance and mean daily diuretic dose (expressed in mg/kg/h both for continuous infusion and for intermittent boluses) were also recorded. Need for dialysis, length of mechanical ventilation, and LOA were documented for all patients as outcome measures.

AKI was defined according to the pediatric RIFLE (pRIFLE) score (acronym of Risk for renal dysfunction, Injury to the kidney, Failure of kidney function, Loss of kidney function, and End-stage renal disease), recently proposed for AKI diagnosis and classification in children based on glomerular filtration rate and UO criteria [13]. Hypokalemia was defined as a potassium level below 2.6 m*M*. Metabolic alkalosis was defined as a pH above 7.45 and bicarbonate concentration above 29 *M* or a base excess level above +5. Whole-blood NGAL was measured by using a point-of-care immunoassay (Biosite Inc., San Diego, CA, USA).

VIS was calculated as follows: dopamine (μg/kg/min) + milrinone (μg/kg/min) × 10 + epinephrine (μg/kg/min) × 100 + vasopressin (UI/kg/min) × 10,000 [14]. CI was measured with the Pressure Recording Analytical Method (PRAM) [15]. This is a minimally invasive hemodynamic monitoring system based on mathematical analysis of the invasive arterial waveform recorded at a high sampling rate (1,000 Hz). The area under the pressure wave is examined during the whole cardiac cycle, including the postdicrotic notch phase, and it is used to assess the patients’ stroke index. PRAM monitoring requires both an arterial waveform without artifacts and the correct identification of the dicrotic notch (it cannot be used in cases of under- or overdamping). The PRAM monitor automatically records each minute’s CI and makes it available for download.

### Statistical analysis

Intention to treat was applied, and all enrolled patients were considered for statistical analysis at the end of the study. The χ^2^ test was used to compare categoric variables. The Student *t* test was used to compare continuous variables. Two-way analysis of variance for repeated measures was used to compare continuous variables over time between the two groups, with the Bonferroni *post hoc* test for each time point. All data are presented as mean and standard deviation (SD). A *P* value <0.05 was considered significant. Statistical analysis was performed by using the GraphPad Prism 5.0 software package (Graph-Pad Software, San Diego, CA, USA).

The study was powered on the primary outcome based on previously available institutional data. The number of patients needed to detect a 1-ml/kg/h difference in UO between the two groups at POD0, with an 80% statistical power and an alpha error of 0.05, was calculated to be 35 for each study group. We planned to enroll a total of 76 patients to accommodate dropouts. An interim analysis was planned after the enrollment of 40 patients, and a stopping rule was determined in case of major morbidity being detected in this phase.

The institutional review board of the Bambino Gesù Children’s Hospital approved the protocol [Prot n. 623RA]. Informed consent was obtained from both parents. The Furocrynic trial was recorded at Clinicaltrial.gov [NCT01628731].

## Results

### Demographic and other study data

Seventy-six patients were enrolled. Two patients were excluded from the EA group after enrollment (and not included in the analysis). The first was excluded because the child was enrolled after 60 minutes from PCICU admission; the second one was excluded because the parents withdrew the informed consent after enrollment. Data from 74 patients were analyzed: 38 in the furosemide (F) group and 36 in the ethacrynic acid (EA) group. The groups were adequately matched for demographic and baseline values (Table 1). Average (SD) age was 104 (180) days in the F group and 135 (181) days in the EA group (*P* = 0.57). Average Aristotle score was 9.5 (2.5) in the F group and 9.7 (2.5) in the EA group (*P* = 0.53). Average VIS at baseline (PCICU arrival) was 22 (18) in the F group and 21 (11) in the EA group (*P* = 0.76). Average fluid overload (excluding bleeding and post-CPB transfusions) was not significantly different in the two populations: 6% (eight) in the EA group and 4% (five) in the F group (*P* = 0.45).

**Table 1:** **Demographic and baseline (PCICU admission) data**

	**Furosemide**	**Ethacrynic acid**	***P***
Number of patients	38	36	-
**Demographic**			
Age (days)	104 (180)	135 (181)	0.57
Weight (kg)	4.6 (3.9)	4.2 (3.1)	0.63
Body surface area (m^2^)	0.27 (0.16)	0.25 (0.11)	0.46
Correction/palliation	11/38 (27%)	10/36 (28%)	0.96
Neonates	18/38 (45%)	17/36 (47%)	0.99
Aristotle score	9.5 (2.5)	9.7 (2.5)	0.53
CPB (min)	187 (70)	204 (102)	0.43
OFF pump (*n*)	5/38 (12.5%)	2/36 (6%)	0.26
XClamp (minutes)	102 (56)	100 (75)	0.88
OFF XClamp (*n*)	4/33 (12%)	2/34 (6%)	0.67
DHCA (*n*)	7/29 (24%)	7/32 (22%)	0.83
**Baseline (PCICU admission)**			
Heart rate (beats/min)	162 (20)	154 (32)	0.25
Syst art press (mm Hg)	77 (13)	81 (19)	0.35
LAP (mm Hg)	6.9 (2.6)	7.5 (1.9)	0.41
CVP (mm Hg)	9.5 (2.9)	9.5 (2.8)	0.98
CI (L/min/m^2^)	2.5 (0.5)	2.8 (0.8)	0.19
VIS	22 (18)	21 (11)	0.76
Cerebral NIRS (%)	54 (18)	57 (16)	0.50
Renal NIRS (%)	68 (20)	69 (19)	0.88
sCr (mg/dl)	0.58 (0.20)	0.57 (0.17)	0.78
CysC (mg/dl)	1.08 (0.4)	1.16 (0.3)	0.58
NGAL (pg/ml)	89 (57)	93 (69)	0.78
pH	7.45 (0.1)	7.45 (0.08)	0.78
HCO_3−_ (*M*)	25 (3.6)	26 (2.5)	0.55
BE	1.29 (4.8)	1.88 (3.3)	0.57
Lactates (m*M*)	5.6 (3.9)	6.3 (3.2)	0.46
SaO_2_ (%)	92 (11)	88 (18)	0.27
SvO_2_ (%)	60 (21)	57 (16)	0.47
PaO_2_/FIO_2_	192 (149)	141 (124)	0.15

The study infusion durations were initially similar in the two groups: from PCICU admission to the end of POD0, the F group received the continuous infusion for 13 (2) hours, whereas the EA received the study drug for 12 (3) hours (*P* = 0.08). Continuous infusion was interrupted for clinical reasons before the end of the study period in 11 F patents and in 23 EA patients (*P* = 0.001): in particular, at the end of POD0, all enrolled patients were still receiving continuous infusions in both groups, whereas at the end of POD1 and POD2, eight and 14 EA patients, respectively, and three and nine F patients were switched to diuretic boluses administration. The reasons for interruption of continuous infusion, as reported by attending clinicians, were polyuria (50%) and excess of negative fluid balance (50%), with no difference in the two groups (*P* = 0.8). All patients who interrupted the continuous infusion received the study diuretic (in boluses, at a lower daily dosage) for the whole 72-hour study period. The diuretic administration was never discontinued as a result of suspected adverse reactions in any patient at any time point.

### Results on renal function

**Figure 1:**
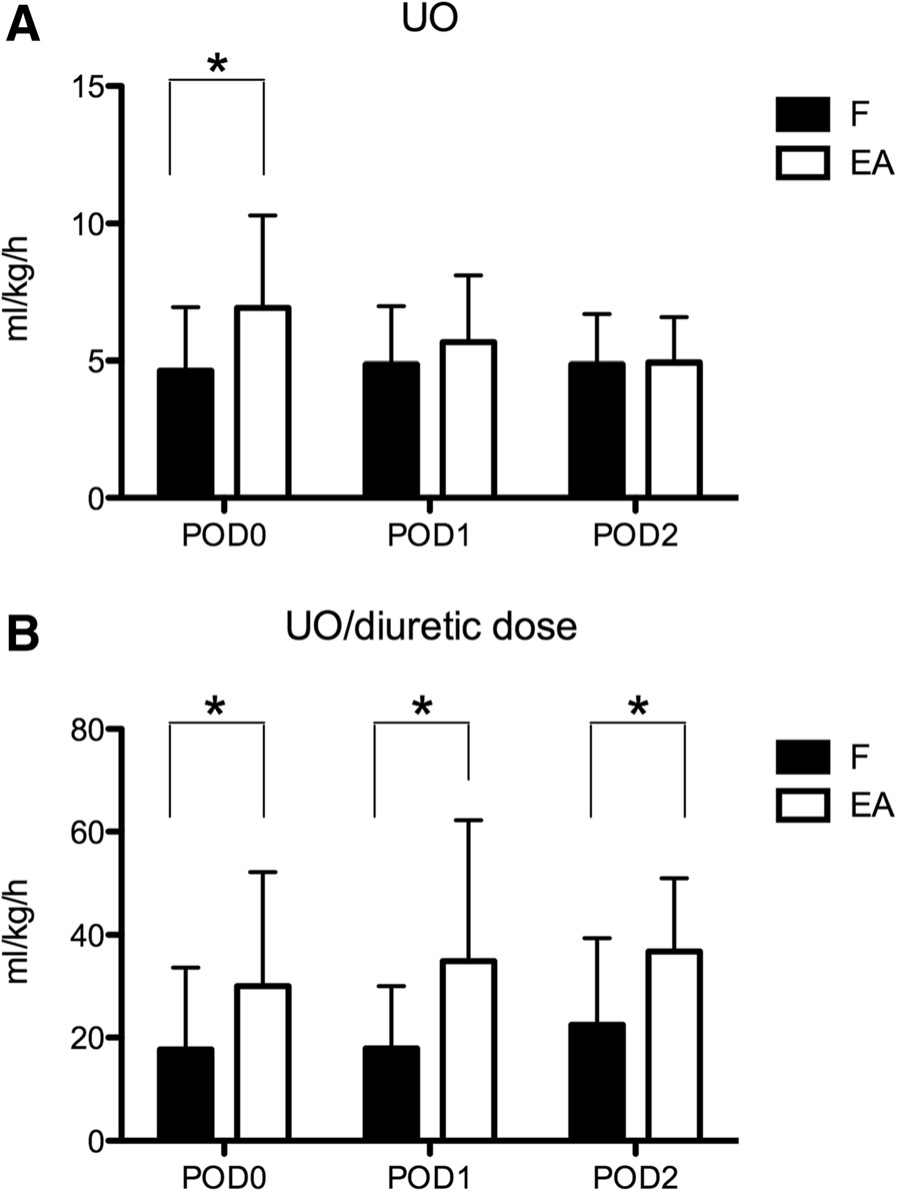
**Urine output (UO) levels expressed in ml/kg/h in the furosemide (F) and ethacrynic acid (EA) groups.** In **(A)** Absolute UO levels.are depicted. In **(B),** UO indexed on diuretic dose are indicated. *P < 0.05. POD, postoperative day. Data are expressed as average and standard deviation.

**Figure 2:**
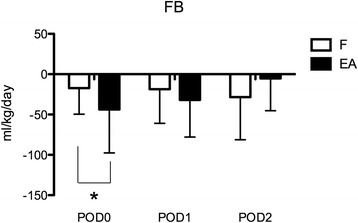
**Fluid balance (FB) per kilogram of patient body weight in the three study days in the furosemide (F) and ethacrynic acid (EA) groups.** *P < 0.05. POD, postoperative day. Data are expressed as average and standard deviation.

The primary end point (UO at POD0) was 6.9 (3.3) ml/kg/h in the EA group, which was significantly higher than 4.6 (2.3) ml/kg/h (*P* = 0.002) in the F group. UO in the following days tended to be similar in the two groups, without significant differences (Figure 1A). However, mean diuretic dose was significantly different in the two groups throughout the study period: overall, mean administered furosemide was 0.33 (0.19) mg/kg/h, whereas mean administered EA was 0.22 (0.13) mg/kg/h (*P* < 0.0001): it can be assumed that for a similar UO, about 30% less EA was needed. A daily diuretic dose above 0.4 mg/kg/h was administered to three patients in the L group (two of these received 0.8 mg/kg/h for 24 hours, and one received 0.5 mg/kg/h for 48 hours) and to one patient in the EA group (0.5 mg/kg/h for 24 hours). The mean UO levels indexed over mean diuretic dose were significantly different in the two groups at every time point (<0.01) (Figure 1B). Fluid balance was significantly more negative in the EA group at POD0: −43 (54) ml/kg/h compared with −17 (32) ml/kg/h (*P* = 0.01) in the F group. Thereafter, fluid balance was similar in the two groups (Figure 2).

**Figure 3:**
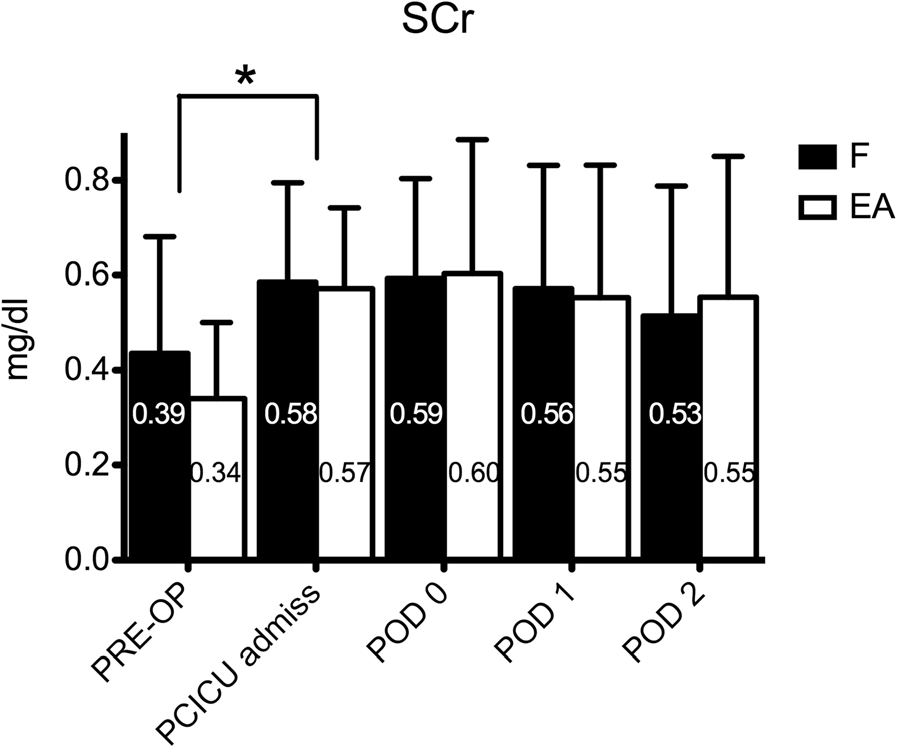
**Serum creatinine (SCr) levels in the furosemide (F) and ethacrynic acid (EA) groups: although significant increase was evident from preoperative (PRE-OP) to Pediatric Cardiac Intensive Care Unit admission (PCICU admiss) levels, no difference was evident between the two study groups over time.** *P < 0.05. POD, postoperative day. Data are expressed as average and standard deviation.

Renal function was apparently unaffected by the use of either diuretic. SCr levels only changed significantly with respect to preoperative values (*P* = 0.0001). However, differences between the two groups were not significant (*P* = 0.54) (Figure 3). CysC and NGAL levels showed similar levels in the two groups over time, without significant differences overall. NGAL average levels were 102.9 (10.87) in the F group versus 112.7 (13.4) ng/ml in the EA group (*P* = 0.25). CysC average levels were 1.2 (0.1) mg/dl in the F group versus 1.3 (0.1) mg/dl in the EA group (*P* = 0.09). Overall incidence of AKI according to pRIFLE was 73% in the F group and 82% in the EA group (*P* = 0.31). The incidence of Injury level (according to pRIFLE) was 35% in the F group and 41% in the EA group (*P* = 0.58). No patient in either group was diagnosed with Failure level or required dialysis. Creatinine at PCICU discharge was 0.35 (0.14) in the F group and 0.34 (0.14) in the EA group. Average percentage difference with baseline creatinine values was −22% (25) and −22% (19) in the F and EA groups, respectively (*P* = 0.99).

### Results on other outcomes

No arrhythmic episode linked to electrolyte disorders was recorded during the study period. Hypokalemia was common: in total, 91 episodes in the F group and 88 in the EA group were recorded in the whole population during the entire study period. Among those who experienced at least one episode of low potassium levels, hypokalemia occurred 2.5 times per day in both groups (*P* = 0.9). Overall, no significant difference was noticed between the percentage number of patients free of hypokalemic episodes during each of the three study days between the F and the EA group (POD 0, 35% versus 44%; POD 1, 33% versus 32%; POD 2, 40% versus 26%; *P* = 0.83). Metabolic alkalosis was also common; 70% of F group patients and 74% of EA group patients experienced at least one metabolic alkalosis event during the study period (p = 0.35). Mean bicarbonate level increased significantly during the first days of the study (p = 0.0001), and appeared to be slightly although significantly higher in the EA group: 27.8 (1.5) mmol/L in the F group versus 29.1 (2) mmol/L in the EA group (p = 0.006). CI was significantly different between the two groups considering that mean VIS at PCICU admission was similar (p = 0.29). Throughout the three study days, it appeared to be improved in the EA group with respect to the F group (p = 0.0081): mean CI values were 2.6 (0.1) L/min/m^2^ in the F group compared to 2.98 (0.09) L/min/m^2^ in the EA group. Length of mechanical ventilation was 5.5 (8.8) days in the EA group compared to 6.7 (5.9) in the F group (p = 0.06). PaO2/FIO2 at the end of POD 0 (when all patients were still ventilated) was similar: 179 (113) in the F group versus 160 (115) in the EA group (p = 0.49). LOA was shorter in the EA group: 14 (19) in the F group versus 16 (15) in the EA group (p = 0.046). No patient died during the study period.

### Results on diuretic dose

ES group was composed by 11 patients, of which 8 from EA group and 3 from F group, whereas 7 children were in the LS group (3 in the EA group and 4 in the F group). ES received a significantly lower amount of diuretic: 0.14 (0.09) vs 0.49 (0.17) mg/kg/h (p = 0.0001). ES patients were significantly bigger, had lower Aristotle score and significantly lower VIS, a significantly lower CPB duration, a significantly lower length of mechanical ventilation, an insignificantly lower LOA (Table 2). Interestingly, whereas UO and creatinine values did not show significant differences between the two groups over time, UO/diuretic dose was significantly different, displaying about double values with respect to LS at all the time points (p = 0.0006) (Table 2).

**Table 2:** **Early stoppers (ES) vs late stoppers (LS) of loop diuretic continuous infusion**

	**ES**			**LS**			**p**
**Pts number**	11			7			-
**Diuretic dose (mg/kg/h)**	0.14 (0.09)			0.49 (0.17)			0.0001
**Weight (kg)**	8.3 (7.7)			3.4 (0.8)			0.03
**Aristotle score**	10 (2)			11 (2)			0.09
**VIS**	19 (10)			23 (9)			0.04
**CPB (min)**	218 (83)			322 (142)			0.04
**LMV (days)**	3 (2)			20 (31)			0.02
**LOA (days)**	11 (18)			24 (25)			0.1
	**POD0**	**POD1**	**POD2**	**POD0**	**POD1**	**POD2**	-
**UO (ml/kg/h)**	5.9 (2.7)	4.1 (2.1)	3.9 (1.9)	6.2 (6.1)	6.1 (3.4)	6.1 (1.1)	0.08
**Creatinine (mg/dl)**	0.5 (0.3)	0.5 (0.25)	0.5 (0.2)	0.6 (0.2)	0.6 (0.3)	0.6 (0.3)	0.17
**UO/diuretic dose**	38.8 (34)	41.5 (25)	40.0 (13)	14.5 (16)	20.2 (20)	11.4 (3.4)	0.0006

## Discussion

Current guidelines recommend using the minimum dose of LD required to keep the patient free of signs and symptoms of congestion [16]. Although the once-daily use of furosemide might be convenient for some patients, it is not optimal from a pharmacodynamics perspective, since daily dosing results in a long period of sodium avidity by the kidney when therapeutic diuretic concentrations are not present. More frequent furosemide dosing serves to limit this “rebound” effect [17]. The use of continuous infusion LD has attracted tremendous attention. This method has proven beneficial in patients with a diuretic resistance or tolerance to conventional intermittent therapy [18]. Several potential advantages of continuous infusion LD have been identified, including decreased electrolyte loss secondary to a lower dosage of diuretic, production of a more reliable urine flow, and decreased alterations in fluid balance [19]. Luciani et al. [11] found that the administration of LD via continuous infusion produced a more controlled diuresis with less variation in hemodynamic parameters.

Furosemide has been the most widely studied loop diuretic with regard to administration via continuous infusion in children [20]. In a study by van der Vorst et al. [2], the authors suggested that continuous high-dose intravenous (IV) furosemide was well tolerated, safe, and effective in reducing volume overload in hemodynamically unstable infants after CPB surgery.

Ethacrynic acid has been studied in pediatric patients in a single IV dose form and in multiple intermittent IV doses [21-25]. These studies used a dose of 1 mg/kg/dose. To date, only one retrospective study has evaluated the use of ethacrynic acid continuous infusion for >12 hours in pediatric patients: 9 patients (including only one neonate) received prolonged EA continuous infusion at a dose range of 0.1-0.3 mg/kg/h [26]. However, ethacrynic acid is considered to be 30% less potent than furosemide, and is associated with a greater incidence of ototoxicity [12].

The importance to control fluid balance and to limit fluid overload in post operative cardiac surgery patients has been clearly demonstrated in several retrospective studies that have associated fluid overload to suboptimal outcomes (in terms of cardiac, pulmonary and renal function) [4-7]. Fluid balance can be managed in such patients by reducing administration of fluids (which is barely possible in the immediate post operative phase due to the high fluidic requirements for intravascular replacement and vasoactive drugs administration) or by increasing UO.

With this background, it is evident the importance of identifying the LD that produces the greatest UO at the lowest dose with, possibly, reduced side effects and renal injury.

Our findings establish a greater effect of EA on UO and fluid balance on POD 0. During the following study days, being the clinicians free to modify the diuretic dose according to UO, EA dose was decreased significantly quicker than the F group: at the end, a 30% lower EA dose was needed to achieve a similar UO in the two groups on each of the study days. Finally, EA was more frequently interrupted than F likely due to a more frequent achievement of negative fluid balance or polyuria as explicitly stated by the attending physicians. According to these results, EA in infants appeared to be about 30% more potent than furosemide. The administration of aggressive diuretic therapies has generated concerns about renal safety, and some controversial results have been published in recent years [27,28]. In our patients, both types of LD, administered at high continuous infusion for 24 to 72 hours, appeared to be safe in terms of renal function. SCr, CysC, and NGAL levels did not show different behaviors in the two groups. Creatinine increased significantly from preoperative level to PCICU admission as previously described [29]. Although preoperative CysC and NGAL values were not available (collection of these biomarkers started for the purpose of exclusive study only after enrollment, at PCICU admission), their post-operative trends were not significantly different in the two groups, and they never reached truly pathologic levels. AKI incidence according to pRIFLE was quite high in the two groups even if pRIFLE classes were not significantly different for the two groups. We can speculate that this was probably related to the high-risk population of small patients undergoing complex surgery; the high AKI occurrence may be also ascribed to the high sensitivity of pRIFLE (previous studies determined an incidence of 50-60% AKI in small cardiac patients) [30-32]: furthermore, about half of these patients had mild renal dysfunction, whose association with worse outcomes has been recently questioned [33]. Even if this study was not designed in order to determine the association between the dose and the renal function, it cannot be excluded that the incidence of AKI might be associated to a high diuretic dose. It should be also acknowledged that the continuous infusion of the two study drugs was allowed to be increased up to 0.8 mg/kg/h, although this is a higher dose then what generally described elsewhere: this was done secondary to institutional policy in the absence of previous literature recommending the maximum continuous infusion doses of LD. However, only 4 patients over a total of 74 (5%) required an average dose higher than 0.4 mg/kg/h and none of them presented short terms side effects linked to diuretic high dose. As a matter of fact, in order to verify if an association between diuretics and renal function we attempted a secondary analysis comparing children who received a low diuretic dose (during a relatively short period of continuous infusion) with those who received a higher dose (and continued the continuous infusion during the whole study period): this small secondary analysis showed that higher doses of infused diuretic were associated to the complexity of surgery (smaller patients with higher vasoactive requirements and longer CPB) rather than renal function as evidenced by similar creatinine values in the two groups. Finally, creatinine levels at PCICU discharge were not increased with respect to baseline values, likely showing that short-term high dose diuretics did not have long-term effects on renal function. Interestingly, the clinical effects on POD0 seem to warrant improved hemodynamics in EA patients, since the CI remained slightly though significantly higher, even if it should be acknowledged that CI showed a baseline insignificant tendency to be higher in the EA group. An improved ventricular performance seems likely in patients achieving timely negative fluid balance [5]. The same benefit was identified at the lung level, justifying a shorter mechanical ventilation time and length of PCICU admission. Unsurprisingly, more than two-thirds of our patients experienced at least one episode of hypokalemia or metabolic alkalosis during the evaluated period. Interestingly, whereas incidence of decreased potassium levels was similar in the two groups, EA displayed a significantly higher potential for causing metabolic alkalosis. This was probably related to its higher effects on UO and the contraction of children volemia. This finding is in line with the small report by Miller and coworkers who recorded an incidence of metabolic alkalosis of 55% after administration of EA [26]. Hypochloremic metabolic alkalosis is a common complication of LD therapy due to the loss of extracellular fluid and chloride [34] and it is probably overlooked in the setting of neonatal/infant critical illness: its effects would merit attention in future studies.

### Limits of the study

This study has some strengths and some limitations. For the first time, we evaluated furosemide effects on UO compared to EA. The children in the study were a rather homogenous population in terms of age and type of surgery. In particular, we evaluated a cohort of high-risk patients (average Aristotle score above 9) with a high incidence of (mild) post-operative AKI. Furthermore, the enrollment occurred soon after PCICU admission from the operatory room in order to avoid that the patients received one or more diuretic boluses before the continuous infusion and to optimize matching of the compared groups. In light of this, decision to start diuretic continuous infusion was left to the attending clinician, which is a rather unspecific inclusion criterion. However, this was made deliberately because the choice of starting continuous infusion was not changed during the study period with respect to institutional everyday clinical practice. The intraoperative fluid balance at PCICU admission was computed to evaluate if significant differences were present between the two populations: this was only an approximation because it did not include post CPB transfusions and bleeding and it likely underestimated the actual degree of fluid status of our patients. Our cohort displayed a relatively high length of PCICU admission: when considered as a whole, average LOA of our patients was 13 days (12) and median LOA was 9 days (5–17). Our results are in line with the “early FO” group of Hassinger study (median LOA 9 days (5.75–12.3) [5] and with those detailed in the higher fluid overload tertile of Seguin’s study (average LOA of 11.7 days (7.9)) [6]. Another important limitation of this trial is the relatively small number of patients studied. This was due to the primary outcome that did not require a particularly large sample. It is possible that with a greater number of patients, some of the secondary objectives, especially those related to adverse effects (namely, metabolic alkalosis), would have been better evaluated. Furthermore, we did not evaluate the long-term effects of short-term high diuretic doses, such as ototoxicity. These might be a subject of further study in the future. To our knowledge, however, clinically relevant long-term adverse effects such as damage to hearing function are limited to a small percentage of critically ill patients receiving long-term courses of (ototoxic) drugs. It is unlikely that short-term LD continuous infusion would significantly contribute to long-term clinical sequelae.

## Conclusions

In a relatively small cohort of high-risk cardiac surgery infants, EA produced more UO compared to F on POD0. Generally, less EA dose is required to achieve similar UO than F. EA and furosemide appeared to be equally safe in terms of renal function, but EA caused a more intense metabolic alkalosis. Finally, EA patients benefited from improved urine flow and optimized fluid balance in terms of better CI, shorter mechanical ventilation, and PCICU admission time.

## Key messages

- EA produced more UO compared to F on POD0. Generally, less EA dose is required to achieve similar UO than F.
- Ethacrynic acid and furosemide at a continuous infusion dose from 0.2 to 0.8 mg/kg/h appear to be equally safe in terms of renal function.
- Ethacrynic acid is responsible for a more intense metabolic alkalosis.
- In our study, improved fluid balance warranted by ethacrynic acid in the first post-operative day revealed improved average cardiac output levels and, probably, shorter duration of mechanical ventilation and Pediatric Cardiac Intensive Care Unit stay.

## Abbreviations

AKI: Acute kidney injury
CHD: Congenital heart disease
CI: Cardiac index
CPB: Cardio-pulmonary bypass
CysC: Cystatin C
EA: Ethacrynic acid
F: Furosemide
LD: Loop diuretics
NGAL: Neutrophil gelatinase associated lipocalin
PCICU: Pediatric Cardiac Intensive Care Unit
POD: Post-operative day
PRAM: Pressure recording analytical method
pRIFLE: Pediatric RIFLE
sCr: Serum creatinine
SD: Standard deviation
UO: Urine output
VIS: Vasoactive inotropic score
